# PAX3: A Driver of Normal Development and Disease

**DOI:** 10.3390/biom16030450

**Published:** 2026-03-17

**Authors:** Noah B. Prince, Joyce H. Liang, Theresa M. Rosato, Deborah Lang

**Affiliations:** Department of Dermatology, Boston University, Boston, MA 02118, USA; nbprince@bu.edu (N.B.P.); jhrliang@bu.edu (J.H.L.); tmrosato@bu.edu (T.M.R.)

**Keywords:** PAX3, melanocyte, melanoma, neural crest, PAX proteins, transcription factor

## Abstract

PAX3 plays a vital role in regulating proper growth, migration, differentiation, and survival during development of normal tissues, including those derived from the embryonic neural crest. PAX3 is a transcription factor with two separate DNA-binding domains and can positively (and less frequently, negatively) regulate gene expression. The levels of PAX3 can be modified by upstream molecular pathways, and its subsequent downstream functions are regulated through a wide range of protein interactions and posttranscriptional modifications. PAX3 direct downstream target genes are other transcription regulators and factors that modulate cellular proliferation, lineage specificity, migration, and survival. The pathways that PAX3 regulates during development may be recycled and subverted during disease progression, for example, during cancer progression, growth, and metastasis. Indeed, PAX3 is overexpressed in several cancers, including melanoma, neuroblastoma, and rhabdomyosarcoma. While there is still much that is unknown about the mechanisms by which PAX3 controls such a wide array of key cellular functions, a great deal of progress has been made to advance our understanding of this critical and multi-faceted factor.

## 1. Introduction

Paired Box 3 (PAX3), also known as HUP2, WS1, WS3, or CDHS, is a transcription factor that plays important roles in both normal development and pathogenesis. PAX3 is a member of the 9-gene PAX family, which is characterized by the highly conserved paired box domain but with greater variations in other regions of the protein between family members [1]. PAX3 also contains a homeodomain, and the interplay between these two DNA-binding domains allows PAX3 to bind a wide range of DNA sequences to regulate transcription of many vital genes, especially during development. This complexity is further expanded by PAX3’s ability to cooperatively bind other factors to enhance transcription of target genes, increasing the number of interactions PAX3 can participate in to regulate gene expression.

Given the large number of possible interactions and downstream targets of PAX3 activity, PAX3 is subject to a high degree of transcriptional regulation itself. As a gene that is primarily expressed during development in normal states, PAX3 lies downstream of several prominent developmental pathways. It is transcriptionally promoted by effectors of the Hippo and Wnt/β-catenin pathways and repressed by effectors of the Sonic hedge hog (Shh) and TGFβ pathways. Through the interactions of these opposing pathways, PAX3 expression is maintained in the neural crest, central nervous system, and somites during development, before being downregulated in most tissues as cells undergo terminal differentiation.

During embryogenesis, PAX3 directs the expression of a large number of genes that influence the development of the tissues in which it is active. PAX3’s target genes are best characterized in the developing neural crest, melanocytes, and myocytes. In these tissues, PAX3 promotes the expression of genes that influence differentiation, proliferation, and migration, and is essential for proper development.

PAX3 mutations and improper expression often lead to developmental abnormalities, homozygous loss, embryonic lethality, and other serious implications [2,3]. Mutations that impede PAX3’s DNA-binding ability are found in Waardenburg syndrome, a family of developmental disorders that can manifest in cell types that rely on PAX3 during development, namely melanocytes and cells of the nervous system. PAX3 is also strongly implicated in melanoma development. While PAX3 is not often mutated in melanoma, it is frequently overexpressed, driving the expression of genes that regulate survival, migration, invasion, and proliferation. Therefore, there is a recapitulation of many of the roles PAX3 promotes in a developmental context [4,5]. PAX3 expression may indeed be a core factor for melanoma development and progression, as a block of PAX3 expression results in loss of melanoma cell viability [6]. PAX3 is a dynamic and multi-faceted transcription factor that plays numerous roles in both development and disease, underscoring the importance of improving our understanding of this vital factor.

## 2. PAX3 Structure and Post-Translational Modifications

**Figure 1:**
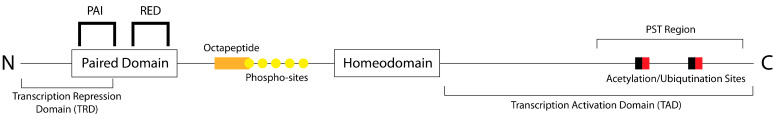
Schematic representation of PAX3 protein. DNA-binding domains are depicted as labeled boxes. The two paired domain subdomains (PAI and RED) are indicated via thick brackets. Other domains of PAX3 are indicated via thin brackets. The orange rectangle refers to the octapeptide motif, the yellow circles represent phosphorylation sites, and the black and red squares represent ubiquitination/acetylation sites. Abbreviations: N, N-terminal end; PAI, N-terminal subdomain; RED, C-terminal subdomain; PST region, proline, serine, and threonine-rich region; C, C-terminal end. The figure was created in Adobe Illustrator 30.2.1.

The canonical PAX3 protein possesses two domains that bind DNA and promote transcription factor function: the highly conserved paired domain (PD) at the N-terminus and a homeodomain (HD) located centrally (Figure 1). The PD comprises two subdomains, PAI and RED, both of which contain a helix-turn-helix motif. When binding DNA, the C-terminal helix of each helix-turn-helix motif makes base-specific contacts along the major groove of the DNA double helix [7]. While PAI facilitates DNA recognition and binding, RED, though capable of DNA binding, is dispensable for protein-DNA interaction [8]. Studies suggest that conformational changes resulting from PAX3 protein–protein interactions alter the DNA-binding affinity of the RED subdomain, yet its precise role remains elusive. The paired domain recognizes the DNA sequences T(T/C)(C/A)(C/T)(G/C)(G/C) and GTCA(C/T)GG, with slight variations seen between the paired domains of other PAX family proteins [9,10,11,12,13]. The PD is also capable of binding other proteins. PAX3 interacts with the transcription factor SOX10 via the paired domain in order to activate transcription of c-RET [14], and can also be bound by calmyrin to inhibit transcriptional activity by inhibiting DNA binding [15]. Through interactions with both DNA and other proteins, the PD serves as a primary regulator of PAX3’s transcriptional activity.

The HD binds DNA and facilitates protein–protein interactions. It contains three major subdomains, each governed by a helix-turn-helix motif. Helical motifs I and II are structural elements of the PAX3 protein HD, while helical motif III is involved in DNA recognition and binding. The HD performs dual roles: first, in binding proteins and cooperating with the PD to activate transcription [8], and second, in DNA recognition, synergizing with the PD for transcriptional activation [16]. The HD is also capable of binding DNA without the contribution of the PD, although Pax3 is likely ineffective in inducing genes with the HD alone [17]. This was further supported by an unbiased screen in melanoma cells, where an analysis of PAX3 binding sites found that HD-only binding rarely occurred [9]. The HD binds the sequences TAAT(N)_2–3_ATTA, T(A/G)AT(T/C)GATT(A/T), ATTA(N)_8_GTTAT, AAT(T/C)(A/G)ATT(A/T), and TAAT(T/C)A [9,18,19,20,21,22]. Like the RED subdomain of the PD, the HD is not required for the binding to DNA, although the involvements of the PD and HD enable the recognition of more specific nucleotide patterns. When the PD and HD bind DNA together, they can recognize the sequences GT(C/T)A(C/T)(G/A)(C/G)N_2_(A/G)TTA and (C/G)(C/T)(G/A)TG(A/G)(C/A)(T/A)AAT [9,20,23]. Structural studies of PAX3 have provided evidence that the protein can adopt a conformation in which the HD can interact with DNA proximally to DNA bound by the PAI subdomain of the PD during DNA binding to dual PDHD sites [10,24,25]. In doing so, this multi-domain cooperation increases PAX3’s binding affinity for target DNA sequences compared to binding by just PD or HD alone [10]. While there are likely further complexities to the interactions between PAX3’s domains that have yet to be identified, the ability of PAX3 to bind DNA with and without the contribution of one or more domains underscores the dynamic and flexible nature of PAX3’s ability to bind DNA.

While the main known role of PAX3 is DNA binding through the PD and the HD, there are several other domains and motifs within PAX3 that serve to modify transcriptional activity; this allows for different protein–protein interactions that give PAX3 a great deal of functional flexibility in regulating gene expression. Nested between the PD and the HD is a short linker region which is not fully characterized, although previous studies have identified two motifs that are important in the regulation of PAX3 activity. First, the linker region contains an octapeptide motif with the sequence HSIDGILS [26]. This motif is known to bind to and recruit transcriptional repressors, such as calmyrin, DAXX, and the groucho ortholog GRG4 [15,27,28]. Second, the linker region contains serines that can be phosphorylated at three major sites: serine 201, serine 205, and serine 209 [29,30,31]. PAX3 also has potential phospho-sites at serines 193 and 197, although no clear evidence of phosphorylation of these amino acids has been documented [31]. Serine 205 phosphorylation by CK2 has been suggested to be necessary to prime serine 201 for phosphorylation by GSK3 in myocytes [30], although other work suggests that GSK3 was able to phosphorylate both of these targets by itself, at least in melanoma cells [31]. For serines 201 and 209, there is no well-defined outcome of phosphorylation. For serine 205, there is a correlation with a proliferative and undifferentiated state, as serine 205 phosphorylation is detected in proliferating myoblasts, but this modification is lost upon differentiation [29,30]. PAX3 phosphorylation in rhabdomyosarcoma cell lines improves DNA binding and transcriptional activation, although the precise phospho-sites were not identified [32]. Phosphorylation by GSK3 may also be important for PAX3 stability, as GSK3 inhibition was shown to decrease levels of PAX3 protein in multiple melanoma lines [31]. Through protein interactions and phosphorylation, the linker region serves as an important regulator of PAX3’s transcription factor function.

The final structural elements of the PAX3 protein are the N-terminal transcription repression domain (TRD) and the C-terminal transcriptional activation domain (TAD). The TRD is located within the first 90 N-terminal amino acids of the PAX3 protein, which includes the first 57 amino acids of the paired domain [33]. The TRD was shown to downregulate transcriptional activity of the N-CAM promoter in a reporter system, although the same study failed to show an interaction between wild-type PAX3 and the N-CAM promoter [33], underscoring the need for further study to better understand the function of this domain. The TAD, on the other hand, is within the 78 C-terminal amino acids of Pax3 [33,34]. It is a region enriched in prolines, serines, and threonines, similar to the TADs of other transcription factors such as OCT-2 and CTF-1 [33]. Functionally, it is likely that the TAD helps to stabilize the paired domain and homeodomains on DNA targets [35]. Ultimately, while both the TRD and TAD have been identified within PAX3, neither is well understood and both would be well served by further study.

While the TAD is primarily known for its role in activating transcription, there are also sites within it that can be post-translationally modified to regulate PAX3 activity. Lysines 437 and 475 can be monoubiquitinated by Taf1, allowing for binding by RAD23B and subsequent shuttling to the proteasomal protein S5a for degradation [36]. This interaction was discovered in developing muscles, where PAX3 is initially expressed but downregulated as muscle cells differentiate, mirroring the expression pattern seen in other developing tissues in which Pax3 is downregulated during terminal differentiation [37,38,39]. In addition to ubiquitination, lysines 437 and 475 can also be acetylated. PAX3’s acetylation status modifies its transcriptional activity, as removal of these acetyl groups increases promoter activity of Hes1 and decreases promoter activity of Neurog2, two Pax3 transcriptional targets during neuronal development [40]. Interestingly, despite the divergence in promoter activation, Pax3 acetylation appears to be necessary for efficient binding in both Hes1 and Neurog2 promoters. The answer to this apparent contradiction is the transcription factor deacetylase SIRT1, which colocalizes and interacts with Pax3 on the Hes1 and Neurog2 promoters on embryonic chick day 9.5, but not on day 12.5, allowing for earlier activation of Hes1 and later activation of Neurog2, which correlates with their respective roles in neuronal development [40]. Acetylated Pax3 also binds the TGFβ2 promoter alongside HDAC1 and p300/CBP to stimulate transcription [41]. Taken together, these studies of Pax3’s lysines 437 and 475 demonstrate an additional layer of flexibility in controlling the activity of PAX3, either by promoting its degradation when it is no longer needed or altering its transcriptional activity during specific developmental timepoints.

## 3. Regulation of PAX3 Expression

**Figure 2:**
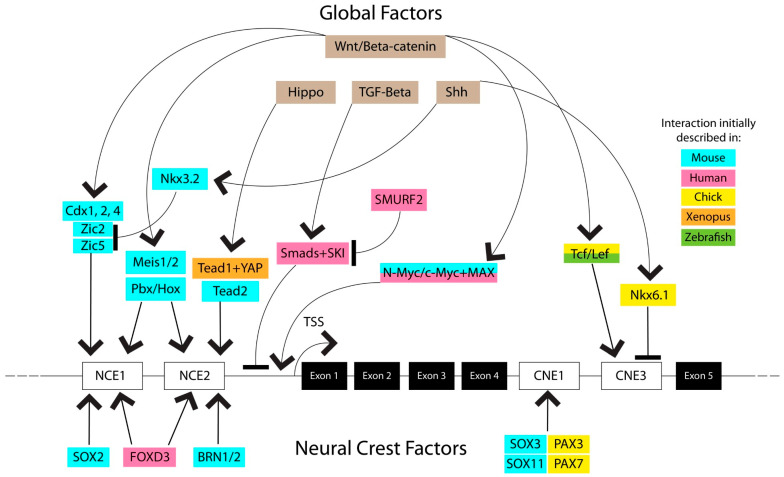
Schematic representation of enhancer regions that regulate the *PAX3* gene. Proximal regulatory elements and the transcription factors that bind them are depicted, as well as upstream regulators of those transcription factors. Factors interacting with the regulatory elements are color coded based on the species in which the interaction was initially discovered. Global factors that regulate gene expression are placed above the *PAX3* regulatory region, while neural crest-specific factors are placed below. The figure was created in Adobe Illustrator 30.2.1.

There are three known regulatory elements responsible for PAX3 transcription. The most distal identified element is an enhancer 7 kb upstream of the *pax3* transcription start site (TSS) that plays a role in the development of hypaxial somite lineages, though this element is the least characterized of the three [42]. The second element is a 1.6 kb enhancer region upstream of the TSS, which is divided into “neural crest elements” (NCEs) 1 and 2 [3,43,44]. The third element is located between the 4th and 5th exons of the *pax3* gene, and is divided into “conserved non-coding elements” (CNEs) 1 and 3 [45]. The interplay between these three regulatory elements, especially the NCEs and CNEs, regulates the transcription of Pax3 (Figure 2).

The NCEs contain binding sites for several transcription factors that regulate Pax3 expression. Both NCE1 and NCE2 possess binding sites for the transcription factors Brn1, Brn2, Meis1, Meis2, and Pbx/Hox family members [43,46]. While Brn2, HoxA1, or HoxA3 individually were insufficient to alter *pax3* expression, co-expression of Brn2 and HoxA1 (but not Brn2 and HoxA3) could drive Pax3 gene activity [43]. Additionally, while HoxB4 is capable of driving *pax3* activity individually, *pax3* activation was increased when HoxB4 was paired with Pbx1 or Meis1 [46]. In melanoma, FOXD3 has also been shown to bind NCE1 and NCE2, and increased FOXD3 subsequently elevated PAX3 expression [47]. However, FOXD3 knockdown did not affect PAX3 expression, indicating that FOXD3 is sufficient but not necessary to drive PAX3 expression in that context. The transcription factors Tead2 and Tead1 bind NCE2 and act as *pax3* transcriptional activators [3]. Tead1 also recruited the transcriptional coactivator YAP, and this complex drove Pax3 expression in developing *xenopus* [48]. Further studies of *pax3* regulation have identified another source of regulation in the Wnt/β-catenin pathway. Wnt expression induces production of Cdx factors, and Cdx1, 2, and 4 bind NCE2 alongside Zic2 and Zic5 to promote *pax3* expression [49,50]. Additionally, a Sox protein binding site was found in NCE2, and co-expression of Sox2 alongside Cdx1 and Zic2 was shown to induce expression in an NCE2 reporter system more strongly than any of the three factors individually or any combination of two [50]. NCE2 is also a site for indirect repression of *pax3* expression. Shh expression upregulates Nkx3.2, which in turn transcriptionally suppresses expression of *pax3* [51,52]. This is accomplished by repressing the expression of Zic2 and Zic5, thereby preventing them from promoting *pax3* expression [50]. Surprisingly, given the array of regulators that bind the NCEs, this region is not necessary for PAX3 expression, as deletions of NCE1 and 2 in a mouse model result in normal development and normal Pax3 expression during development [3], which suggests a level of redundancy of the *pax3* enhancers.

To understand the apparent inessentiality of the NCEs, it is necessary then to look to the CNEs, as the intronic CNE1 and 3 (also called ECR2) are sufficient to drive *pax3* expression. Studies of the CNEs have found binding sites for Tcf/Lef factors in CNE3, which are also downstream effectors of Wnt signaling [3,45]. CNE3 also contains a binding site for Nkx6.1, overexpression of which can reduce Pax3 expression in chick embryos [45]. While CNE3 contains binding sites for both positive and negative regulators, CNE1 appears to be more geared towards positive regulation, showing evidence of binding by Sox3, Sox11, Pax3, and Pax7 [45]. Sequence comparisons across species show that the CNEs are more heavily conserved than NCEs, suggesting that CNEs are the primary regulatory region for *pax3*, with NCEs functioning as a redundant region that protects *pax3* expression from inactivation due to mutations in the enhancers [45]. After initial CNE3 activation by Wnt signaling, it is possible that CNE1 is then responsible for maintenance of Pax3 expression through its Pax and Sox factor binding sites, since Pax3 expression is still maintained after Wnt signaling subsides [45]. Taken together, these results suggest high-level regulation of *pax3* by opposing action of Wnt and Shh expression. Downstream Wnt factors such as Cdx, Tcf/Lef, and Hox genes bind sites in the NCEs and CNEs to stimulate transcription, while Shh downstream factors Nkx6.1 and Nkx3.2 suppress PAX3 expression either directly through these enhancers or indirectly through Zic2 and Zic5. In the context of development, these two master regulatory pathways act in opposition to each other to produce the correct pattern of Pax3 expression in the developing neural crest.

Outside of these identified regions of PAX3 regulation, there are sites for binding by Smad factors between NCE2 and the transcriptional start site of *PAX3* [53]. Their binding can be induced by TGFβ signaling, and they function to reduce *PAX3* transcription. This Smad complex is also bound by SKI, although the exact outcome of that interaction is unclear [53]. SKI has been reported in melanoma as a corepressor in TGFβ signaling through its ability to bind Smads and prevent them from activating transcription, with knockdown of SKI resulting in increased PAX3 expression [54,55]. On the other hand, SKI has been reported as an important driver of melanoma, knockdown of which slows melanoma growth [56]. In melanoma, this repressor complex is counteracted by SMURF2, an E3 ubiquitin ligase that can downregulate Smad proteins and rescue PAX3 expression [57].

One of the Smad factor binding sites proximal to the PAX3 transcriptional start site also overlaps with a binding site for the Myc-Max family of transcription factors [53,58]. In contrast to the effects of Smad binding, N-Myc and c-Myc promote PAX3 expression. N-Myc and c-Myc are capable of activating PAX3 transcription by themselves, although activation is increased when they are accompanied by Max, which is incapable of driving expression on its own [58]. c-Myc expression is promoted in turn by Wnt/β-catenin signaling, following the trend of Wnt effectors promoting the expression of PAX3 [59,60]. These interactions outside of the more regularly studied NCEs and CNEs underscore the many layers of complexity that go into controlling the expression of PAX3.

## 4. PAX3’s Known Transcriptional Targets and Binding Partners in Normal States of Development

PAX3 is expressed in specific cell types during development, including myogenic precursors, the nervous system, and the neural crest (NC) and its derivatives [61]. While other reviews extensively cover other PAX3 lineage tissues, here we will focus on NC, melanocytes, and myocytes, cell populations in which PAX3 target genes are well characterized.

### 4.1. PAX3 in the Developing Neural Crest

Neural crest cells, originating between the neural plate and non-ectoderm, are multipotent embryonic stem cells important in vertebrate development. Located at the border of the neural plate, NC cells undergo extensive differentiation and migration to give rise to a diverse set of cell and tissue types, including melanocytes, neural glial cells, cartilage, bone, and connective tissues [39]. Pax3 emerges as a key regulator orchestrating the migration, differentiation, and survival of NC-derived cell lineages [62]. PAX3 is essential for the proper development of NC, with heterozygous and homozygous PAX3 mutations often leading to birth defects and embryonic lethality [2,3]. During embryonic development, Pax3 expression precedes NC differentiation, peaking during midgestation before gradually declining upon completion of differentiation [63].

PAX3 regulates several genes that have a range of effects on NC development. One factor that is crucial in normal development is WNT1. In PAX3-expressing tissues, Wnt1’s proximal 5′ promoter is bound by Pax3, promoting expression [64]. Wnt signaling has an important role in the development of numerous cell types, so it is likely regulated by multiple factors across the tissues in which it operates. In the NC, Wnts are involved in the initial induction of the NC and differentiation into specific NC lineages [64]. Further, PAX3 plays a role in directing differentiation in the developing NC. One example of this is enteric ganglia, where loss of Pax3 led to significant neural deficiencies in the gut. It was discovered that Pax3 cooperates with the transcription factor SOX10 to drive c-RET expression, a tyrosine kinase receptor which is required for normal development of the enteric ganglia [14,65]. PAX3 also regulates factors that drive NC cell migration such as CXCR4 and MSX2. PAX3 partners with the transcription factor FOXD3 to drive CXCR4 transcription and facilitate NC cell migration, which is a crucial function given the extensive migration NC cells need to undergo to reach all of their target tissues during development [66,67]. Pax3 expression also suppresses the expression of Msx2, a Bmp effector that negatively regulates the migration of cardiac NC cells, thereby promoting NC cell migration [68]. Pax3 also interfaces with Bmp signaling by promoting the expression of SOSTDC1, an inhibitor of BMP signaling [69]. This functions to repress Bmp-induced differentiation of cranial NC into osteogenesis, highlighting the role of Pax3 as a repressor of terminal differentiation. Similarly, Pax3 drives the expression of the proneural transcription factors Hes1 and Ngn2. In the absence of Pax3, and by extension Hes1 and Ngn2, NC cells show reduced migration and premature neurogenesis, leading to severe defects in spine and brain development [40]. PAX3 is essential in the proper development of the NC, particularly in regard to cell migration and proper timing of differentiation.

### 4.2. PAX3 in Melanocytes

In developing melanocyte precursors (melanoblasts), PAX3 functions as an important regulator, binding to promoter sequences of downstream melanocytic genes such as MITF and the melanin pathway genes TYRP1 and DCT, while exerting regulatory control over cell survival, proliferation, differentiation, and migration [14,27,70,71]. MITF, known as a master regulator of melanogenesis, is a particularly important target of PAX3, as it promotes the transcription of several melanocyte-specific genes, including TYR, TYRP1, and TYRP2/DCT [72]. Pax3 also interacts with another transcription factor, Sox10, and facilitates transcriptional activation of multiple targets [14,27]. The differentiation of NC cells into NC-derived melanocytes is closely linked to the Wnt1 signaling pathway. Under non-differentiating conditions when the Wnt1 pathway is not active, Pax3 forms a transcriptionally active complex with Sox10 that binds to the Mitf gene promoter [14,27]. During differentiation, Mitf protein binds to promoters of several melanocyte factors, including the downstream Dct promoter region [27]. Without a robust Wnt signaling response, Pax3 joins a repressor complex with Grg4 and Lef1 and displaces Mitf from a Dct enhancer region [27]. In the presence of pro-differentiation Wnt signaling, increased cytoplasmic β-catenin levels disrupt the interaction between Pax3 and Lef1, thereby inhibiting the formation of a repressor complex. Instead, β-catenin-Lef1 complexes with Mitf and Sox10 and promotes differentiation by facilitating binding to the Dct gene promoter and driving transcription of downstream genes [27,73,74]. Pax3 also drives transcription of Tyrp1, a gene that is important for melanogenesis and melanin production, a core functionality of melanocytes [71,75]. PAX3 is also a regulator of another Wnt/β-catenin responsive transcription factor, BRN2. PAX3 binds the BRN2 promoter, and both it and β-catenin are capable of driving BRN2 transcription [76,77]. BRN2 also has a similar expression profile to PAX3 in developing melanocytes, with higher expression earlier in development that decreases as melanoblasts differentiate into melanocytes [78]. While the exact role of BRN2 in developing melanocytes and neural crest is not fully defined, in melanoma it is highly correlated with an invasive phenotype [79,80], so its function is likely related to regulating migration. In melanoma cells PAX3 has also been shown to bind BRN2 and cooperate with it to regulate MITF transcription, although there are conflicting reports as to whether this regulation is positive or negative [79,81,82]. The expression of PAX3 in developing melanocytes regulates differentiation, proliferation, migration, and melanogenesis, often operating downstream of and/or in cooperation with Wnt/β-catenin signaling.

### 4.3. PAX3 in Myocytes

Unlike the major cell types derived from the NC that we discussed here (peripheral nervous system, melanocytes, etc.), only a small minority of muscle cells are derived from the NC. The majority of myocytes are derived from the embryonic structures called somites, where PAX3 is also expressed and has an important function in the proper development of muscle tissue. In mammals, PAX3/Pax3 is expressed prior to somite segmentation and within the pre-somite paraxial mesoderm [83,84,85,86]. As the somite forms, Pax3 (as well as paralog Pax7) is expressed throughout. As the somite matures and partitions dorsally into the dermomyotome and the sclerotome ventrally, Pax3 becomes confined within the dermomyotome structure [63]. A lack of Pax3 expression in the dermomyotome results in cells failing to thrive [87,88,89,90]. Matching what is seen with maturing NC cells, Pax3 expression is downregulated as cells migrate from the dermomyotome in parallel with the promotion of myogenic differentiation.

The progression of somite precursor cells to skeletal myogenic lineages involves genetic cascades involving several genes. Upstream genes Msgn1, Meox1/2, and Six1/4 are important in early specification, and deficiencies lead to *Pax3* impairments [91,92,93]. However, in mouse embryonic stem cells and mouse embryos, Pax3 was determined to be a main upstream driver and determinant of myogenic lineage specificity by remodeling the chromatin of muscle-specific enhancer regions [94]. Within these developing myocytes, Pax3 occupies enhancers with active histone marks (H3K4me1 and H3K27Ac) and associates with the chromatin looping complex protein LIM-domain binding protein 1 (LDB1) [95]. Reduction in LDB1 attenuates myogenic differentiation, while forced recruitment promotes gene expression and downstream myogenesis. These studies support that Pax3 is key in the regulation of molecular pathways involved with myogenic lineage specification.

As in the general neural crest and melanocyte contexts, PAX3 in developing myocytes has several targets that help to orchestrate proper differentiation. Pax3 drives expression of Fgfr4, Met, Dmrt2, Myf5, and MyoD, all of which are involved in promoting myogenesis [11,96,97,98,99,100,101,102]. Interestingly, Myf5 is downstream of Dmrt2 and upstream of MyoD, so Pax3 cooperates with the products of its transcriptional targets to drive expression of further downstream targets, potentially as a mechanism to ensure proper timing of expression. This is a parallel with PAX3 transcriptional promotion of MITF in melanocytes, where PAX3 also regulates MITF downstream genes. Several myogenic structural proteins were upregulated in Pax3 gain-of-function experiments, and the cell adhesion molecule M-cadherin is a direct target of Pax3 [103,104]. PAX3 also interacts with FOX transcription factor family members during myogenesis. Pax3 cooperates with Foxo3, where the two factors collaborate to recruit RNA polymerase II to form a pre-initiation complex on a *myoD* enhancer [105]. This contrasts with what is seen with Foxc2 (and to a similar degree paralog Foxc1). During development, both Pax3 and Foxc2 are co-expressed throughout the somite, but as the somite matures Pax3 is expressed in the dermomyotome and Foxc2 is primarily located in the sclerotome, with low levels of expression overlap [106]. Pax3 gain of function leads to downregulation of Foxc2 and promotion of a myocyte phenotype over an endothelial cell fate [107]. There is good support that Pax3 and Foxc2 can act as a myocyte/vascular cell switch, which is toggled by Notch signaling [108,109]. Pax3 likely drives expression of Sprouty1, a factor that inhibits terminal differentiation [99], mirroring behavior in other tissues where PAX3 promotes lineage specificity while inhibiting terminal differentiation. This may function to maintain a multi-potent population of precursor cells in developing tissues. In addition, PAX3 drives the expression of the anti-apoptotic genes BCL-XL and TGFβ2, helping to improve survival of developing myocytes [41,110]. As in many other tissues, Pax3 expression is downregulated as development progresses [97], but early Pax3 expression is essential for normal myogenesis and survival of developing myocytes.

## 5. PAX3 in Mature Tissues

While PAX3 expression is crucial in normal development, it plays a smaller role in mature cells and tissues. In contrast with the wide expanse of expression during development across much of the developing neural tube, PAX3 expression in developed tissues is more limited. As neural crest-derived cells differentiate into neuronal, glial, and several other cell types, Pax3 expression is downregulated [37,38,39]. Skin is a rare site of post-developmental PAX3 expression, as PAX3 is expressed in melanocytes and melanocyte stem cells in the skin and within hair follicles [27,111]. In the case of melanocyte stem cells, it is hypothesized that Pax3 inhibits terminal differentiation, helping to maintain a multipotent population of stem cells that are able to respond to environmental stimuli [27].

PAX3 may fill a similar niche in adult muscle as it does in adult skin. Pax3 is downregulated in most adult muscle cells but is maintained in satellite cells, a population of cells that resides alongside muscle fibers and which is involved in postnatal growth and muscle regeneration [112,113]. Satellite cells express Pax7, the member of the PAX family that is closest in structure to Pax3, and a subset also co-express Pax3 [113,114]. The numbers of satellite cells expressing Pax3 vary widely by anatomic location, with the highest numbers in the trunk and forelimb muscles and less in hindlimbs, and are generally absent from cranial musculature [113,115,116]. While Pax7 is observed to be universally expressed in all satellite cells, there are examples of Pax3 expressing and Pax7 negative cells within ex vivo models [117]. Pax3 is not fully redundant to Pax7 in the satellite cells, however. Pax3 positive satellite cells are more resistant to certain environmental pollutants, such as dioxins, suggesting a specialized role in remodeling and repair after injury [113]. Further, there is support that satellite cells expressing only Pax7 are more closely associated with committed myogenic differentiation, while satellite cells maintaining Pax3 expression are more closely associated with a less differentiated phenotype [118].

Finally, the mature nervous system also maintains a small population of PAX3-expressing cells. Pax3 expression was detected in young adult mouse brains in a population of Bergmann glial cells in the cerebellar cortex [119]. Another study detected expression of Pax3 in a small subset of non-myelinating Schwann cells in the sciatic nerve of adult mice [120]. These Pax3-expressing cells also express Sox2 and p75Ngfr, signals associated with multipotency [120]. While additional experimentation would be needed to confirm this hypothesis, it may be that PAX3 expression is maintained in populations of glial cells to allow for a rapid repair response in the event of damage, mirroring the role played by PAX3 in adult melanocyte and muscle satellite cell populations.

## 6. PAX3, Lineage Specificity, and Chromatin Interactions

Although it is not known how PAX3 drives lineage specificity, or why there is specific cell fate differentiation between PAX3-dependent cell types of the neural crest, somites, and other tissues, one potential mechanism is that PAX3 acts as a pioneer factor in regulating the expression of lineage-specific genes. Pioneer factors interact with silent heterochromatic regions to promote activation or to further repress these regions. The recruitment of cell-type-specific cofactors by pioneer factors promotes lineage-specific gene expression and differentiation, while a loss of pioneer factors leads to an inability of a cell to differentiate (reviewed in [121]). The ability of PAX3 to act as a pioneer factor has not been directly tested and proven, but there is compelling evidence that supports this possibility: PAX3 remodels chromatin [94], promotes the expression of lineage-specific genes [122], and reprograms cells toward alternate cell fates [102]. PAX3 also interacts with HP1, which induces H3K9me3 heterochromatin compaction and gene silencing, thereby actively silencing these regions [123]. Further, the related factor PAX7 has pioneer functions, and the similarity between these proteins implies some shared functions [124,125,126].

Most of the clues suggesting that PAX3 acts as a pioneer factor are from works on myocytes and myogenic precursor cells. The induction of PAX3/Pax3 expression led to changes in chromatic accessibility and gene expression pathways that promoted a muscle cell fate [94]. Cell environment plays a critical factor, with the introduction of Pax3 into non-somitic/myoblast precursor cells, such as mouse fibroblast 3T3 cells, leading to chromatin remodeling but not to muscle-specific gene expression. PAX3 may prime loci by promoting a poised state, where pioneer factors are bound to enhancers, but the chromatin maintains a closed conformation. Similar behaviors have been observed for other pioneer factors, including Foxa2, GRHL proteins, and the PAX3-related protein Pax7, which can prime genomic regions but are unable to open chromatin and drive gene expression alone [125,127,128]. Activation of these enhancers occurs through the recruitment of other transcription factors and may be driven by known PAX3-transcriptional partners, such as SOX, ETS, or FOX proteins. At least with myogenic programming, PAX3-promoted cell fate programming involves chromatin looping with distal bound elements and interacts with chromatin looping proteins including subunits of cohesin and LDB1 [95]. Examples of these distal enhancers include MYF5 and DMRT2 (Table 1). In terms of LDB1, interaction with Pax3 induces myogenesis, and inhibition of LDB1 blocks myogenic differentiation [95]. While it is not clear how PAX3 promotes cell fate decisions, it is likely due to priming lineage genes and recruiting or inhibiting transcriptional partners to regulate gene expression. However, PAX3’s potential function as a pioneer factor requires further investigation.

## 7. PAX3 in Waardenburg Syndrome

Given the importance of PAX3 expression in the proper progression of neural crest development, it is unsurprising that dysregulated PAX3 expression can cause developmental disorders. Aberrant PAX3 expression is seen in human Waardenburg syndrome types I and III, genetic disorders affecting approximately 1 in 212,000 individuals [130]. These syndromes manifest with diverse phenotypes, including hearing impairments, pigmentation anomalies, and central nervous system abnormalities. First described in 1951 by ophthalmologist and geneticist Petrus Johannes Waardenburg [131], Waardenburg syndrome (WS) is commonly associated with hypopigmentation, though some also experience moderate to profound hearing loss in one or both ears [132]. This loss of hearing is due to cochlear melanocyte dysfunction. Of the four clinical subtypes, type I and type III involve direct loss-of-function mutations of the *PAX3* gene. There are over 50 different ways these mutations can manifest, including frameshift, nonsense, splice site, point mutations, and even entire gene deletions [133,134]. A direct result of these mutations is often a loss or reduction in expression of genes that are downstream of PAX3, such as WS type 1-associated PAX3 mutants being incapable of driving MITF or TYRP1 expression [70,71]. While not every PAX3 mutant in Waardenburg syndrome has been characterized, several studies have identified mutations in the PD or HD, some of which were further shown to have impaired DNA-binding capacity, which would seriously inhibit PAX3’s ability to induce transcription of target genes [19,135]. Phenotypic presentations of type I typically involve congenital sensorineural hearing loss, dystopia canthorum of the eyes, neural tube defects, cleft lip or palate, and patchy depigmentation of the skin and hair [136,137]. Type III is extremely similar to type I, though often more severe, and additionally includes musculoskeletal abnormalities. Both subtypes are inherited in an autosomal dominant pattern, but type I is the most common, whereas type III is the rarest [138]. Clinical manifestations are the result of insufficient neural crest cell migration and differentiation during embryogenesis, emphasizing the role of PAX3 in these processes.

## 8. PAX3 in Melanoma

**Figure 3:**
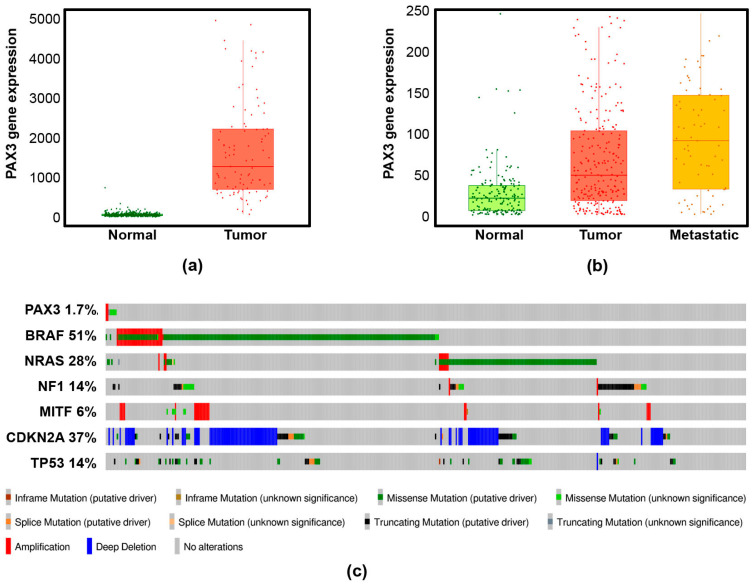
PAX3 is overexpressed but rarely mutated in melanoma. (**a**). Plot of PAX3 expression in normal skin vs. melanoma from the TNMplot RNA-seq database [141]. Significance was determined with a Mann–Whitney test. (**b**). Plot of PAX3 expression in normal skin vs. primary melanoma vs. metastatic melanoma from the TNMplot genechip database [141]. Significance was determined with a Kruskal–Wallis test. (**c**). cBioPortal analysis of mutation rates in various melanoma driver genes in the TCGA cutaneous melanoma dataset [144].

PAX3 also plays an important role in melanoma. Melanoma is the deadliest form of skin cancer, and while advances in treatment over the last few decades have improved survival rates (15.7% 5-year survival rate for metastatic melanoma from 1992–2005 vs. 29.8% 5-year survival rate by 2021), it remains a serious concern [139,140]. PAX3 is regularly overexpressed in melanoma, and its expression is negatively correlated with melanoma survival (Figure 3a,b) [4,5,6,141]. In other cancers, such as rhabdomyosarcoma, PAX3 can become fused with other transcription factors such as FOXO1 through chromosomal translocations [142,143], but this is a rare event in melanoma (Figure 3c). Indeed, in contrast to other melanoma drivers, PAX3 mutations in melanoma are highly uncommon (Figure 3c) [144], indicating that PAX3 is capable of driving melanoma activity mainly through overexpression of the native protein. PAX3 appears to drive melanoma by recapitulating some of its developmental roles in this new context, particularly in promoting migration, proliferation, and survival.

PAX3 is known in melanoma for transcriptionally driving the expression of genes that are associated with migration, such as BRN2 and CXCR4. Like PAX3, these factors are expressed during development and downregulated during differentiation but overexpressed in melanomas [76,78]. Depletion of PAX3 upstream of BRN2 can reduce melanoma cell migration [76], while direct depletion of BRN2 in a xenograft mouse model showed a decrease in metastatic tumor growth [80]. Depletion of PAX3 and/or FOXD3, both of which drive CXCR4 expression, causes decreases in melanoma cell motility, migration, and chemotaxis, while exogenous expression of CXCR4 rescues this phenotype [66]. By promoting these factors, PAX3 promotes melanoma cell migration, a crucial contributor to invasion and metastasis.

PAX3 also regulates the expression of genes involved in promoting melanoma progression. One example of this is MET, which is driven by PAX3 and SOX10 binding cooperatively to the MET promoter [129]. Inhibition of MET causes a reduction in melanoma proliferation and an increase in apoptosis, and MET knockdown also decreases melanoma proliferation [145]. MET expression increases in parallel to the stage of the melanocytic lesion (nevus, primary tumor, metastatic melanoma), and mice with overactivated Met signaling have increased rates of melanoma formation [145,146]. Increased MET activity was also found in a melanoma cell line that was resistant to vemurafenib, a commonly used melanoma chemotherapeutic that targets BRAF^V600E^ oncoprotein [147]. This resistance was overcome by MET inhibition or knockdown, as a combination of those treatments with vemurafenib treatment led to a decrease in melanoma proliferation, invasion, and migration [147].

MITF expression, which is also driven by a cooperative interaction between PAX3 and SOX10, is another factor that plays multiple roles in driving melanoma activity [14]. In vitro suppression of MITF expression in melanoma reduces proliferation, migration and invasion, while in vivo MITF suppression reduces rates of metastasis and reduces tumor size [80]. TP53 expression is also increased in melanoma cells following MITF suppression, suggesting that MITF is involved in promoting melanoma survival [80]. By promoting the expression of MET and MITF, PAX3 promotes melanoma proliferation, migration, invasion, and survival.

PAX3 can also promote melanoma activity through its opposition to apoptosis induced by the classical tumor-suppressor p53 through a MITF-independent pathway. PAX3 is essential for melanoma cell survival, as transient knockdown significantly diminishes cell viability [6]. This is at least partially due to an inverse relationship between PAX3 activity and apoptotic events, with PAX3 knockdown leading to increases in active caspase-3 and P53 protein, demonstrating its role in apoptosis inhibition [148,149]. Surprisingly, further study of this dynamic showed that PAX3 expression is inversely correlated with p53 protein, but not RNA levels, suggesting that PAX3 regulates p53 through a non-transcriptional mechanism [150]. Additional investigation of the Pax3-p53 relationship showed that Pax3 downregulates p53 at the protein level by promoting p53 association with the ubiquitin ligase Mdm2, leading to increased p53 ubiquitination and degradation [151]. A Pax3 variant lacking both the TRD and TAD but containing the PD and HD is still capable of binding both p53 and Mdm2 and triggering p53 ubiquitination, underscoring that this interaction is not transcriptionally dependent. There is also evidence to suggest that PAX3 impedes the transcriptional activity of p53, as PAX3 expression can suppress the transcription of p53 downstream target genes BAX and HDM2, although it is also possible that this result was a byproduct of PAX3-induced p53 degradation and not transcriptional repression by PAX3 [152]. These studies show that while PAX3 plays an important role as a transcription factor, it can regulate gene expression via alternate mechanisms.

## 9. PAX3 and PAX3-Translocation Products in Alveolar Rhabdomyosarcoma (aRMS)

Rhabdomyosarcoma (RMS) is the most common pediatric soft-tissue sarcoma. The most common variants of RMS are embryonal RMS (eRMS), which accounts for approximately 60–70% of patients, and alveolar RMS (aRMS), comprising 20–30% of cases [153,154]. The aRMS tumors primarily contain PAX3 (and to a lesser extent, paralog PAX7) chromosomal translocations, so much so that these genetic abnormalities are now used as a core clinical diagnostic for this RMS type [155]. Of these PAX translocations, the majority involve the FOXO1 gene, with 60% t(2;13)(q35;q14) or 20% t(1;13)(p36;q14), producing PAX3-FOXO1 or PAX7-FOXO1 fusion products, respectively [156,157,158,159,160]. There are other PAX3 translocation products discovered in aRMS, but these are rare. These include gene fusions with the FOXO1-related protein FOXO4, transcriptional coactivators MAML3, NCOA1 and NCOA2, muscle specific coactivator myocardin, and ATP-dependent chromatin remodeling protein INO80D [161,162,163,164]. While PAX3 translocations are a major defining feature of aRMS, no parallel translocations are reported in melanoma. Indeed, our cBioportal analysis did not reveal evidence of any PAX3 translocations in a large melanoma dataset (Figure 3).

The chromosomal region of PAX3 included in the PAX3-FOXO1 fusion protein encodes both the PD and HD but lacks most or all the TAD region (see the locations of these regions in Figure 1). These sections fuse to the C-terminal transactivation domain of FOXO1 [156,157,158,159,160]. The resulting PAX3-FOXO1 fusion protein acts as a stronger transcriptional activator when compared to wild-type PAX3 [165,166,167]. This potency is likely due to the combined function of both transcription factors replicating their roles from normal development [105]. Alternatively, or additionally, the increased transcriptional capacity of PAX3-FOXO1 may be due to a loss of inhibitory protein domains, or loss or gain of regulatory enhancer elements. Genetic amplification of the PAX3-FOXO1 allele is uncommon while transcript levels are frequently significantly higher than wild-type levels [168], supporting a role of altered regulatory regions.

In parallel to what is seen in melanoma with wild-type PAX3, PAX3-FOXO1 promotes cell growth, survival, migration, and lineage specificity while inhibiting terminal differentiation [169,170,171,172,173,174,175]. Ectopic expression of PAX3-FOXO1 alone may not drive these oncogenic cellular phenotypic changes; rather, there is a promotion of myogenic gene expression and growth arrest [176,177]. This switch to a myogenic lineage can also occur in non-myogenic cells, even when wild-type PAX3 fails to do so. An addition of a second oncogene, such as MYC or CyclinD, can overcome the growth arrest and drive pro-tumor cellular functions [177]. Not only does MYC enhance PAX3-FOXO1 oncogenic function, but it is also a downstream effector gene target of the fusion protein [178,179]. The activity and stabilization of both PAX3-FOXO1 and MYC are supported by several enzymes, including PLK1, Aurorah kinase A, and the deubiquitinating enzyme YOD1, as well as PAX3-FOXO1 cofactor BRD4 [180,181,182,183].

PAX3-FOXO1 drives the tumorigenesis by binding to native PAX3 enhancers as well as new sites. The fusion protein can combine the normally separate PAX3 and FOXO1 topologically associated domains (TADs) together to form novel TADs that drive PAX3-dependent and independent gene expression, often with an association with E box sites [172,184,185]. Histone acetylation is also altered by PAX3-FOXO1 through CBP/p300 and RNA polymerase II recruitment [186]. Many of the genes targeted by PAX3-FOXO1 are muscle lineage genes, as well as other genes that are dysregulated in melanoma, including MET tyrosine kinase receptor and CXCR4 [174,187]. PAX3 translocation products are a main feature of aRMS and are a core reason for the aggressiveness of this tumor type. While this fusion protein shares many functions with wild-type PAX3, the oncogenic function of PAX3-FOXO1 is expanded due to a wider array of downstream targets, additional protein partners and modifiers, and a greater transcriptional capacity.

## 10. PAX3 in Neuroblastoma

PAX3 is also dysregulated in neuroblastoma, a cancer of neural crest-derived neuroblast cells. While advances in treatment have improved survival over time, the 5-year survival rate for high-risk neuroblastoma still sits at 50% [188,189]. PAX3 expression has been detected in multiple neuroblastoma cell lines, with higher expression being seen in more malignant cell lines [190]. Knockdown of PAX3 in these cell lines results in impaired proliferation, migration, and survival after treatment with chemotherapy drugs [190]. Overexpression of PAX3 in neuroblastoma cells also increases rates of polysialylation of NCAM [191]. Rates of polysialylation of NCAM, in turn, are positively correlated with proliferation, migration, and invasion in neuroblastoma [192,193,194]. It should also be noted that *PAX3* is transcriptionally promoted by N-Myc, which is regularly overexpressed in neuroblastoma cases [58,188]. While there is not a great abundance of study of PAX3 in the context of neuroblastoma, what data is available suggests that PAX3 plays a similar role as in melanoma, promoting proliferation, migration, invasion, and survival.

## 11. Conclusions

PAX3 is a highly dynamic factor that can drive large changes in gene expression, making it a prime candidate for further study in multiple contexts. This is especially true given the number of questions remaining about PAX3’s function in both development and disease. A prime example of this is the list of PAX3 transcriptional targets included in this review. We elected to list only genes that are well-characterized PAX3 targets, but there are many other genes that have their expression changed by perturbations of PAX3, raising the possibility that PAX3 affects the expression of a much wider array of genes either directly or indirectly. This is compounded by the ability of PAX3 to bind other proteins. It is unlikely that all PAX3 binding partners have been discovered, allowing for further complexity in how PAX3 regulates basic cell functions. This is further underscored by the ability of PAX3 to affect p53 stability via protein–protein interactions instead of transcriptionally. If PAX3 is capable of non-transcriptionally affecting protein dynamics of other targets, that could dramatically expand our understanding of PAX3’s function in regulating gene expression. Finally, the role of PAX3 in cancer development is another area that would benefit from further study. Many factors that are important to melanoma development are associated with a particular melanoma phenotype (invasive or proliferative) and their expression in a given melanoma can vary based on the phenotype of that cancer. PAX3, on the other hand, appears to be overexpressed nearly universally in melanomas (Figure 3), which may indicate a more central role in melanoma biology. Meanwhile, the PAX3 fusion proteins seen in aRMS result in strong transcriptional activators with a widened pool of targets, increasing the complexity of an already nuanced factor. Given the importance of these unanswered questions, further study of PAX3 should be a priority in the fields of developmental and cancer biology.

## Figures and Tables

**Table 1:** PAX3 target genes with proven PAX3 binding sites. NC, neural crest; PD, paired domain; HD, homeodomain; PDHD, dual paired domain homeodomain site; UTR, untranslated region; ND, not determined.

PAX3 Direct Target Genes						
Gene Target	Enhancer Location	Cell Type	Binding Site	Transcription Factor Partners	Regulation	Ref
**General neural crest genes**						
CXCR4	5′ UTR and 1st intron	NC, melanoma	Intron PD, 5′ UTR ND	FOXD3	↑	[66,67]
HES1	5′ proximal promoter	Neural tube/NC	PD/ND		↑	[40]
MSX2	5′ promoter region	NC	PD		↓	[68]
WNT1	5′ proximal promoter	NC	PD		↑	[64]
NGN2	5′ promoter	Neural tube/NC	PD/ND		↑	[40]
RET	5′ enhancer	NC	PD	SOX10	↑	[65]
SOSTDC1	5′ proximal promoter	NC	PD		↑	[69]
**Neural/melanocyte genes**						
DCT	5′ proximal promoter	Melanocytes	PD	Tcf/Lef, Groucho	↓	[27]
MITF	5′ enhancer	Melanocytes, melanoma	PD, PDHD	SOX10, BRN2	↑	[70,71,79]
TYRP1	5′ enhancer	Melanocytes melanoma	PD		↑	[75]
BRN2	5′ proximal promoter	Melanoblasts melanoma	PD		↑	[76]
**Somite/muscle genes**						
DMRT2	5′ distal enhancer (18 Kb)	Somites	PDHD		↑	[96]
MYF5	5′ distal enhancers (57.5, 111 Kb)	Somites myocytes	PDHD, PD		↑	[97]
MYOD	5′ proximal promoter	Myocytes	PD	FOXO3	↑	[98,105]
BCL-XL	5′ proximal promoter	Myocytes	Possible PD or PDHD		↑	[110]
FGFR4	Enhancer 3′ of gene	Somites	PD		↑	[99]
TGFβ2	5′ proximal promoter	Somites	ND		↑	[41]
MET	5′ proximal promoter	Myocytes melanoma	PD	SOX10	↑	[11,100,129]

## Data Availability

Not applicable.

## Abbreviations

The following abbreviations are used in this manuscript:

aRMS	Alveolar rhabdomyosarcoma
C	C-terminal region
CNEs	Conserved non-coding elements
eRMS	Embryonal rhabdomyosarcoma
HD	Homeodomain
LDB1	LIM-domain binding protein 1
N	N-terminal end
NC	Neural crest
NCEs	neural crest elements
ND	Not determined
PAI	N-terminal subdomain of Paired domain
PAX3	Paired Box 3, human protein (gene in italics)
Pax3	Paired Box 3, mouse, xenopus, zebrafish, and chicken protein
*pax3*	Paired Box 3, mouse, xenopus, zebrafish, and chicken gene
PD	Paired domain
PDHD	Dual paired domain homeodomain site
PST	Proline, serine, and threonine-rich region
RED	C-terminal subdomain of Paired domain
RMS	Rhabdomyosarcoma
Shh	Sonic hedge hog
TAD	Transcriptional activation domain
TRD	Transcription repression domain
UTR	Untranslated region
WS	Waardenburg syndrome
